# The impact of clarifying the long-term solution picture through solution-focused interventions on positive attitude towards life

**DOI:** 10.1371/journal.pone.0267107

**Published:** 2022-05-13

**Authors:** Gen Takagi, Kazuma Sakamoto, Naoto Nihonmatsu, Miki Hagidai

**Affiliations:** 1 Faculty of Comprehensive Welfare, Tohoku Fukushi University, Sendai, Japan; 2 Graduate School of Education, Tohoku University, Sendai, Japan; 3 Japan Society for the Promotion of Science (JSPS), Tokyo, Japan; 4 Department of Child Development, Shibata Gakuen University, Hirosaki, Japan; Taipei Medical University, TAIWAN

## Abstract

Solution-focused brief therapy is a psychotherapeutic model. The purpose of this study was to examine the effects of clarity of long-term solutions on positive attitude towards life. In order to examine the effects of the long-term solution image, the conditions for clarifying the long-term and short-term solution images, and not seeking clarification of the solution image were set and randomly assigned. A total of 94 participants who responded to all questions were included in the analysis. The results of this study indicate that clarity of the long-term solution enhances time-oriented attitude. In addition, the clarity of short-term solutions increases the reality of their goals. Furthermore, solution-building and, positive, and ideal levels of life were shown to increase after implementation, regardless of the condition. These results indicate that clarification of the long-term solution expands the positive attitude of valuing limited time.

## Introduction

According to the Occupational Safety and Health Survey performed by the Japanese Ministry of Health, Labour and Welfare [1], 59.5% of workers experienced increased stress levels related to their work and professional life and 0.4% of employees took a leave of absence owing to mental health difficulties. Therefore, preventing stress-related mental health problems has become an urgent issue in Japan. Ebine [2] pointed out that a positive attitude towards life is a useful indicator of favorable aspects of mental health. A positive attitude towards life is the degree to which one is actively living one’s own life. It can be viewed through five aspects: goals and dreams, ambition, positivity, time-orientation, and selfishness [2]. The higher the positive attitude towards life, the higher the subjective happiness and sense of fulfillment that arise [2]. Therefore, it is possible to improve the mental health of workers by increasing positive attitude towards life, which is the topic of investigation in this study.

Solution-focused brief therapy (SFBT) is one way to improve positive attitude towards life. SFBT is a psychotherapeutic model proposed by de Shazer et al. [3]. In SFBT, understanding the problem does not necessarily lead to a solution for the problem [4], and the focus is on solution-building, which centers on clarifying goals and extending exceptions rather than problems. Solution-building improves communication within the organization, empowers the organization, increases efficiency, and improves performance and morale [5]. In addition, therapists who practice SFBT have observed that SFBT affirms clients’ thinking and improves their motivation, confidence, and mood [6]. In particular, positive thinking, high motivation, high self-confidence, and positive mood are central elements of a positive attitude towards life, and SFBT has the potential to enhance them.

Questions from the therapist play an important role in the solution-building process of the SFBT. The premise behind this is that in SFBT, the client is an expert in his or her life and the therapist is an expert on asking questions to help the client achieve the life he or she wants [7, 8]. Therefore, it is important for the therapist to ask questions that allow the client to make use of the helpful information they have. Specifically, the Miracle Question (MQ) was used to clarify goals [9]. The MQ is a guidepost for support because it defines the client’s desired future after the problem has been solved [10]. According to previous research [11], in order to set clear goals, a goal should be (1) important to the client, (2) limited to the situation, specific, actionable, and measurable, and (3) realistic. Such goals make the solution image clearer, and the client’s hope for their future life and their efficacy regarding the problem increases [12]. An exception question (EQ) expands on exceptions, which are defined as times when the problem does not occur or is slightly better. Even the most persistent problems are not always present, and there are some exceptions [13]. When clients begin to look for exceptions, they can find and reproduce exceptions that they had not previously recognized [14]. Solution-focused principles and techniques help clients utilize their inherent, perhaps unconscious thought processes and experiences to find solutions [13].

### SFBT worksheet

SFBT, in which questions play an important role, has been shown to be effective not only in face-to-face support, but also in worksheet-based support. Worksheet-style support removes the constraints of time and place and allows people to work at their own pace [15]. L’Abate [16] pointed out that descriptive workbook practices can efficiently and immediately support multiple participants simultaneously using standardized procedures that can be replicated. This kind of worksheet-based support is both practically and empirically important, as it is an effective means of resolving stressful situations for workers. It is also useful in that it allows the scientific comparison of individual techniques. In previous research [17], a worksheet based on the MQ was developed and its effectiveness was compared with that of a problem-focused worksheet. The results showed that the MQ worksheet increased the degree of problem-solving and self-efficacy compared to the problem-focused worksheet. In addition, a worksheet consisting of the EQ and MQ was developed in a previous study [18], which found that it increased the degree of problem-solving, self-efficacy, and solution-building. The effects of time-machine questions (TMQ) have also been examined in previous research [19].

TMQ is a technique that requires participants to imagine long-term solutions as if they were placed in a time machine, which is part of the SFBT technique developed in a previous study [20] in Japan. Doraemon, one of the most popular manga characters in Japan, can move freely along the time axis using a time machine, which frequently appears in the story. Therefore, the concept of time machines is highly familiar to the Japanese people, and the term "time machine" is an effective way to frame the concept of a long-term future. The TMQ technique is characterized by an extension of the solution’s timeline. While the miracle question asks participants about a solution "tomorrow," the time-machine question attempts to help them imagine a longer-term, future solution. Therefore, time-machine questions tend to be especially useful in cases in which the future is expansive, such as adolescence and young adulthood [20]. Time-machine questions are assumed to have different effects than miracle questions, although no study has quantitatively examined the differences between the two types of questions. In addition, previous research [19] confirmed that TMQ increased positive emotions and decreased negative emotions based on pre- and post-comparisons, but no effect study has been conducted that compared it with other conditions.

### The purpose of this study

The purpose of this study was to examine the effects of worksheets based on the TMQ by comparison, with the aim of proposing solutions to the mental health challenges of workers. A condition asking participants an EQ after clarifying the solution with TMQ and MQ (TMQ+MQ+EQ condition), a condition asking an EQ after clarifying the solution with MQ only (MQ+EQ condition), and a condition asking an EQ without clarifying the solution (EQ condition) were established. To compare the effects of these worksheets, solution-building, positive attitude towards life, and the ideal level of life were measured.

Solution-building can assess an individual’s ability to identify exceptions, solutions, and hopes in the future. Solution-building was measured using the solution-building inventory developed in a previous study [21]. It has been shown that MQ and EQ enhance solution-building [18]; to confirm the additional effects of TMQ, solution-building was measured in this study. In addition, positive attitude towards life scale had five items for "goals and dreams," which is an attitude of trying to have goals and dreams, four items for "ambition," which is an attitude of trying to improve oneself, seven items for "positive," which is an attitude of trying to be positive, six items for "time-oriented," which is an attitude of valuing time, and three items for "selfishness," which is an attitude of trying to focus only on one’s own identity. In particular, the TMQ was expected to increase time-oriented attitudes as it clarified long-term solutions. SFBT is expected to increase goals and dreams, ambition, and positivity because it helps clients pursue goals that they have voluntarily set [22]. Finally, Takagi [23] pointed out that in SFBT, improvements in life, as well as specific problems, may be realized; in fact, the observed tasks of SFBT have been found to increase the ideal level of life. Therefore, since the TMQ may positively change perceptions of life, participants were also asked a single item about the ideal level of life.

## Methods

### Procedure

**Fig 1:**
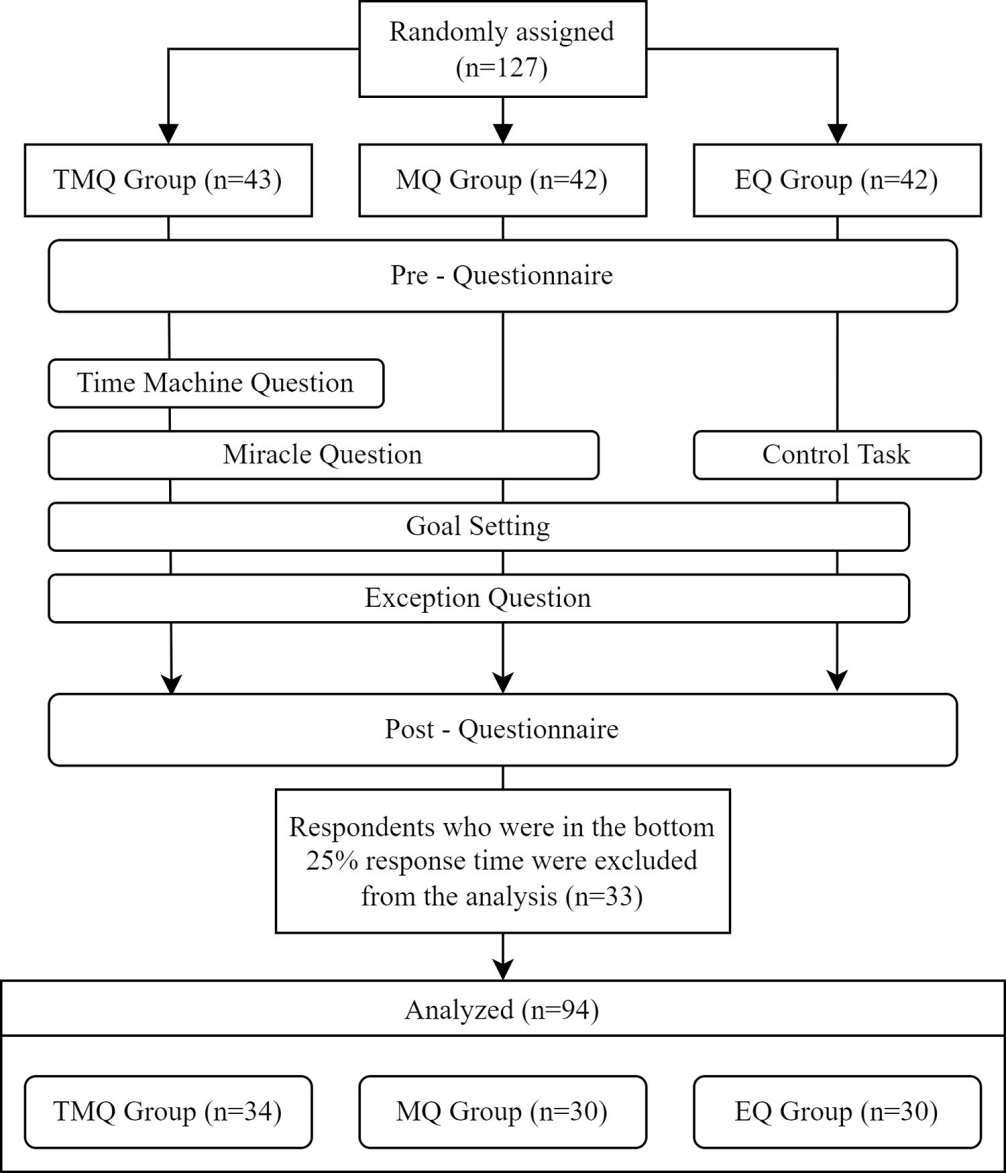
Experimental design.

In June and July 2020, a web-based survey was conducted among working Japanese adults aged between 20 and 39 years, recruiting survey participants through "Lancers," a Japanese company that provides crowdsourcing services (https://www.lancers.jp/). A large variety of people who wish to perform simple tasks are registered on Lancers, and by posting a survey recruitment on Lancers, one can invite people to participate in their survey. In this study, we recruited participants with a reward of 40 JPY. Before being presented with the web-based questionnaire, individuals were asked to participate in the study with an explanation of the contents of the study and the questionnaire form. They were assured that they were not obliged to answer the questions, that their answers were statistically processed, and that their personal information would not be identified. Only those who agreed to participate in this study could proceed to the questionnaire response screen. To compare the effects of TMQ, we randomly assigned the participants to the TMQ condition (TMQ+MQ+EQ group), MQ condition (MQ+EQ group), and EQ condition (EQ group). The pre-effect measures, each condition worksheet and post-effect measures were presented in sequence (Fig 1). In this way, each group responded to the effect measures immediately before and after work.

This survey was conducted after receiving approval from the Research Ethics Review Committee to which the author belongs (approval number: RS200610). As an ethical consideration, at the time of requesting cooperation for the survey, the participants were informed that this survey might ask about their "ideal last day of life." It was also specified that psychological worksheets would be assigned, that cooperation with this study was not compulsory, that responses would be stopped in the middle of the study if they did not want to continue, and that the survey would be anonymous and no personal information would be disclosed.

### Participants

A certain number of participants responded inappropriately to the web-based survey without reading the questions. Previous research [24] showed that when participants were recruited using a crowdsourcing service, 23.7% responded inappropriately without carefully reading the questions. The response time for inappropriate participants was also shown to be shorter than that for appropriate participants [25]. Therefore, to obtain stable results, this study assumed that 25% of respondents did not respond appropriately to the survey; respondents who showed the bottom 25% response time (shorter than 490 seconds) were excluded from the analysis. Finally, 34 participants in the TMQ+MQ+EQ group (19 men and 15women; age range = 23–38 years; mean age = 31.53; *SD* = 4.29), 30 participants in the MQ+EQ group (13 men and 17 women; age range = 23–39 years; mean age = 32.70; *SD* = 5.13), and 30 participants in the EQ group (14 men and 16 women; age range = 21–39 years, mean age 31.93, *SD* = 4.96) were included in the analysis.

### Questionnaire

#### Demographic

Participants were asked about their sex and age.

#### Solution-building inventory

The solution-building inventory developed by Smock et al. [21] can assess an individual’s solution-building ability to identify exceptions, solutions and hopes in the future. A Japanese version of this scale was developed by Takagi et al. [26]. Moreover, Takagi et al. [27] revised the Solution-building Inventory Japanese version into an easy-to-understand Japanese expression. The reliability and validity of the original and Japanese versions of this scale have been confirmed [21, 27]. This scale has a one-factor structure consisting of 14 items such as "I have the ability to focus on what I want to occur in my life," which the participants had to rate on a scale ranging from "1 = strongly disagree" to "5 = strongly agree." The total score of the 14 items divided by the number of items was used as an indicator of solution-building. Cronbach’s alpha coefficient for solution-building was .89, indicating sufficient reliability.

#### Positive attitude towards life

The Positive Attitude Towards Life Scale, which was developed by Ebine [2], validated, and confirmed to be reliable, was used to measure positive attitude towards life, which may be enhanced by the TMQ. This scale has the following five subscales: "goals and dreams" (five items), " ambition" (four items), "positivity" (seven items), " time-oriented" (six items) and "selfishness" (three items). Items are scored on a five-point Likert scale ranging from "1 = disagree" to "4 = agree". "Goals and dreams" is measured by items such as "I’m trying to move towards my dream." "Ambition" is measured by items such as "I’m trying to improve myself every day." "Positivity" is measured by items such as " I’m trying to enjoy every day." "Time-oriented" is measured by items such as "I’m trying to use my time carefully." "Selfishness" is measured by items such as "I’m trying to be myself." The total score of each subscale divided by the number of subscale’s items was used as an indicator of each subscale. Cronbach’s alpha coefficient for "goals and dreams" was .89, "ambition" was .93, "positivity" was .88, "time-oriented" was .88 and "selfishness" was .78. All subscales were above .70 with sufficient reliability indicated.

#### Ideal level of life

To measure the ideal level of life, referring to previous research [28], participants were asked, "regarding your life in general, a score of 0 is given to the severe and worst condition, and if you are living an ideal life, rate it as 10. What score would you give your life now?"

### Structure of the worksheet questions

#### Time machine question

The TMQ+MQ+EQ group was required to answer the TMQ, "Let’s get in the time machine to see the last moments of your life. Please picture in your mind, as if you were watching a scene from a movie about the ideal way to meet the end of your life." To make the future solution specific, we asked for open-ended responses about the season and place, and asked for a choice of three times of the day: morning, noon, and night. They were also asked to respond in an open-ended manner about their thoughts, feelings, and actions at that time. Finally, they were asked to rate, on a scale ranging from 1 (not at all) to 4 (very well), how well they were able to imagine the ideal final moments of their lives.

The TMQ+MQ+EQ group was required to answer the next question, "Let’s get in the time machine to see a moment in time in ten years. Please picture who you wish to be ten years from now." To make the future solution specific, we asked for open-ended responses about the season and place, and asked for a choice of three times of the day: morning, noon and night. They were also asked to respond in an open-ended manner about their thoughts, feelings and actions at that time. Finally, they were asked to rate on a scale ranging from 1 (not at all) to 4 (very well) how well they were able to imagine the ideal moments in time in ten years.

#### Miracle question

The TMQ+MQ+EQ and MQ+EQ groups were required to answer the miracle question, "If a miracle were to happen and tomorrow would be the day you wanted, what would it be like? It would probably differ from the way you live now, and you might feel different when you wake up in the morning. Then, you may do something differently. If the people around you behave the way you want them to, you will probably treat yourself differently as well. Let your imagination run wild and think freely." To make the future solution specific, we asked for open-ended responses about the season and place, and asked for a choice of three times of the day: morning, noon, and night. They were also asked to respond in an open-ended manner about their thoughts, feelings, and actions at that time. Finally, they were asked to rate, on a scale ranging from 1 (not at all) to 4 (very well), how well they were able to imagine the ideal tomorrow.

#### Control task

The EQ group was required to answer the control task, "Take a few moments to reflect on your own current life. Please look back at your current life and write down what comes to mind."

#### Goal-setting

All participants were required to set their goals by responding to the prompt, "Set a goal based on your answer to the MQ. Create a goal that meets the following conditions: The conditions are as follows:

- Action-level goals that are as specific and visible as possible.
- As small and realistic a goal as possible (the first small sign, not the ultimate goal).
- The positive goal of "I will" rather than the negative goal of "I won’t."

They were then asked to rate the concreteness of their goals on a scale ranging from 1 (not at all specific) to 4 (very specific). They were also asked to rate the reality of their goals on a scale ranging from 1 (very difficult) to 4 (very easy).

#### Exception question

All participants were required to answer their exception based on the prompt "Look back on a time when the goals you set were achieved in some measure or when the severity of the problem was a little better. What was different about you at that time that made it so? What can you do to help increase the occurrence of these situations? "

#### Level of commitment to worksheet

We thought that it would not be appropriate to analyze those who did not take the worksheets seriously. Therefore, to determine participants’ commitment to the worksheet, we asked, "How seriously did you work on the worksheet?" Respondents were asked to rate on a scale ranging from 1 (very serious) to 4 (not at all serious). However, no one answered "not very seriously" or "not at all seriously," so all the participants were included in the analysis.

## Results

SPSS (Version 24.0) was used to analyze the data. In all statistical evaluations, a significant difference was considered to exist if the p-value was less than .05. In this study, we assumed a moderate effect and calculated the power for a *η*_*p*_^*2*^ of .06, using GPower (the correlation between repeated measures was set to the default of .50, and the nonsphericity correction e was set to the default of 1.0). The results showed that the power for the main effect of time (pre-score / post-score) was .998, and the power for the interaction between time (pre-score / post-score) and group (TMQ+MQ+EQ group / MQ+EQ group / EQ group) was .994. These results indicate that the power of the analysis was sufficient.

### Descriptive statistics and scale reliability

The average score on each scale was then calculated. The descriptive statistics for each are presented in Table 1.

**Table 1:** Descriptive statistics of scale.

		Pre-Score		Post-Score	
		*M*	*SD*	*M*	*SD*
Solution-building		3.40	0.57	3.51	0.06
Positive attitude	Goals and dreams	3.64	0.70	3.77	0.09
towards life	Ambition	3.52	0.98	3.59	0.10
	Positivity	3.68	0.75	3.81	0.07
	Time-oriented	3.49	0.80	3.57	0.08
	Selfishness	3.48	0.69	4.00	0.07
Ideal level of life		5.50	1.81	5.62	0.18

### Analysis of the effects of TMQ

To compare changes in effect measure scores (solution-building, subscale of positive attitude towards life, ideal level of life) on the worksheet, we conducted a repeated measures analysis of variance (ANOVA) with time (pre / post) as the within-subjects factor and group (TMQ+MQ+EQ group / MQ+EQ group / EQ group) as the between-subject variable. For all statistically significant test results, the partial eta squared (*η*_*p*_^*2*^) was calculated as the effect size. The results of the analysis are presented in Table 2.

**Table 2:** Comparison between the groups of the pre- post-scores by repeated measures ANOVA.

		Pre-Score		Post-Score				
		*M*	*SD*	*M*	*SD*	Time	Time ×group	Simple main effect
Solution-building	TMQ+EQ+MQ	3.47	0.10	3.58	0.10			
	MQ+EQ	3.38	0.10	3.45	0.11	20.36***	0.85	
	EQ	3.35	0.10	3.49	0.11			
Goals and dreams	TMQ+MQ+EQ	3.72	0.14	3.79	0.15			
	MQ+EQ	3.72	0.15	3.79	0.16	3.56	0.37	
	EQ	3.71	0.15	3.73	0.16			
Ambition	TMQ+MQ+EQ	3.51	0.17	3.55	0.17			
	MQ+EQ	3.42	0.18	3.53	0.18	3.07	0.23	
	EQ	3.65	0.18	3.71	0.18			
Positivity	TMQ+MQ+EQ	3.68	0.13	3.86	0.12			
	MQ+EQ	3.64	0.14	3.73	0.13	19.02***	0.58	
	EQ	3.72	0.14	3.85	0.13			
Time- oriented	TMQ+MQ+EQ	3.42	0.14	3.63	0.14			TMQ+MQ+EQ: pre < post***
	MQ+EQ	3.49	0.15	3.51	0.15	6.23*	4.11*	
	EQ	3.55	0.15	3.57	0.15			
Selfishness	TMQ+MQ+EQ	3.90	0.12	4.03	0.12			
	MQ+EQ	3.97	0.13	4.02	0.13	2.89	1.44	
	EQ	3.97	0.13	3.96	0.13			
Ideal level of life	TMQ+MQ+EQ	5.55	0.33	5.71	0.33			
	MQ+EQ	5.40	0.34	5.53	0.33	7.48**	0.41	
	EQ	5.50	0.33	5.57	0.33			

Note

*p < .05

**p < .01

***p < .001.

A repeated measures ANOVA for solution-building showed a significant time effect, *F* (1,91) = 20.36, *p <* .001, *η*_*p*_^*2*^ = .18, indicating that solution-building increases after the implementation of any work; for "time-oriented" showed a significant time effect, *F* (1,91) = 6.23, *p* < .05, *η*_*p*_^*2*^ = .06, and a significant time (pre / post) by group (TMQ+MQ+EQ group / MQ+EQ group / EQ group) interaction effect, *F* (2,91) = 4.11, *p* < .05, *η*_*p*_^*2*^ = .08; indicating that the TMQ+MQ+EQ group had higher time-oriented scores at post compared with pre; and for "positivity" showed a significant time effect, *F* (1,91) = 19.02, *p* < .001, *η*_*p*_^*2*^ = .17, indicating that positivity increased after the implementation of any work. However, a repeated measures ANOVA for "goals and dreams,” “ambition, " and "selfishness" showed no significant effect. A repeated measures ANOVA for the ideal level of life showed a significant time effect, *F* (1,91) = 7.48, *p* < .01, *η*_*p*_^*2*^ = .07, indicating that the ideal level of life increased after the implementation of any of the work.

To identify the effect of TMQ on goal concreteness and reality, an independent-samples t-test was conducted with the presence or absence of TMQ as the independent variable, and goal concreteness and reality as the dependent variable (Table 3). The results showed that there was no significant difference in the evaluation of goals based on the TMQ. Next, to identify the effect of MQ on goal concreteness and reality, an unpaired t-test was conducted with the presence or absence of MQ as the independent variable and goal concreteness and reality as the dependent variable (Table 4). The results showed that the TMQ+MQ+EQ and MQ+EQ groups were rated as more likely to achieve their goals than the EQ group (*t* (92) = 2.45, *p* < .05, *d* = .54).

**Table 3:** Differences in the nature of goals depending on whether the TMQ is implemented.

	TMQ+MQ+EQ (*n* = 34)		MQ+EQ and EQ (*n* = 60)		
	*M*	*SD*	*M*	*SD*	*t*
Goal concrete	1.94	0.65	1.78	0.56	1.25
Goal reality	2.26	0.62	2.43	0.64	1.23

**Table 4:** Differences in the nature of goals depending on whether the MQ is implemented.

	TMQ+MQ+EQ and MQ+EQ (*n* = 64)		EQ (*n* = 30)		
	*M*	*SD*	*M*	*SD*	*t*
Goal concrete	1.77	0.57	1.88	0.60	0.83
Goal reality	2.60	0.50	2.27	0.67	2.43*

Note

*p < .05.

## Discussion

A randomized controlled trial (RCT) experiment was conducted to examine the effects of TMQ, and the results showed that the worksheet consisting of the TMQ, MQ, goal-setting and EQ increased time-orientation. In contrast, solution-building and positive and ideal levels of life increased after implementation, regardless of the content of the work. In addition, evaluations of the set goals were shown to increase with the implementation of MQ and EQ. The effects of the TMQ, MQ, goal-setting and EQ are discussed in detail below.

### Effect of TMQ

Only the TMQ+MQ+EQ group showed an increase in time-oriented scores after implementation, with a medium effect size of *η*_*p*_^*2*^ = .14. Time-oriented refers to an attitude towards spending time in life in a meaningful way, consisting of items such as "I am trying to use my time carefully" and "I am trying to spend my days without wasting them." The focus on long-term solutions through TMQ before implementation of the MQ and EQ increased the participants’ value of time owing to a sense of life’s limitedness and increased their willingness to use their time meaningfully to achieve an ideal solution. TMQ has also been found to increase positive emotions and decrease negative emotions [19]. Although there is concern that facing the finite nature of life may have a negative impact, TMQ dispels this concern by dealing with a positive vision of the future. Therefore, TMQ can be considered a useful technique in that it can simultaneously enhance both positivity and a sense of value for time.

Previous research [2] has shown that the higher the time-oriented perspective, the higher the fulfillment, independence, confidence, trust and time outlook. These are psychological variables related to fulfillment, and as time-orientation increases, fulfillment, confidence, and trust are likely to increase. Furthermore, previous research [2] shows that time-oriented is positively related to fulfillment through solidarity with others. Therefore, time-orientation is associated with a sense of fulfillment that includes both relationships with others and one’s state of being, and it is essential to increase time-oriented qualities through TMQ to increase fulfillment in diverse aspects of life.

### Effect of MQ

The TMQ+MQ+EQ and MQ+EQ groups showed a higher reality for the set of goals than the EQ group. The MQ is a guidepost for support because it clarifies the client’s desired future vision and life after problem-solving [10]. Previous research [29] also reported a more hopeful feeling after responding to the MQ. Furthermore, prior study [17] showed that worksheets structured around the MQ increased problem-solving and self-efficacy. These findings suggest that before implementing EQ, MQ can help people find hope and increase their sense of efficacy by clarifying their desired future vision. The results of this study suggest that not only the efficacy of the problem, but also the efficacy of achieving the goal was increased, and the feasibility of the goal was highly evaluated.

It is also possible to decrease the difficulty of the goal itself. Prior study [30] states that through responses to MQ, participants consider the first step needed to find a solution. Being aware of the first step towards a solution leads to setting a feasible goal. Therefore, it is possible to set a highly feasible goal that would be the first step in the process. Whether the efficacy of these goals has increased or less challenging goals have been found will require further detailed research.

### Effect of goal-setting and EQ

Finally, solution-building was shown to increase after implementation, regardless of the content of the work. Solution-building is a central competency of the SFBT: clarity of goals, extended awareness of exceptions, and expanded hope for the future. In this study, all groups were asked to set goals and respond to the EQ, which may have increased solution-building, including the clarification of goals and increased awareness of exceptions.

Goal-setting and EQ have been shown to increase positivity. The results of this study support the findings of prior research [6] that therapists who practice SFBT observe that it affirms clients’ thoughts and improves their motivation, confidence, and mood. Positivity is an attitude of positive enjoyment of life, consisting of items such as "I try to enjoy every day" and "I try to live with high expectations." In addition, previous research [2] has shown that positivity is related to a sense of fulfillment regarding one’s state. This suggests that goal-setting and EQ are characterized by increased positive enjoyment of life and subjective fulfillment.

Goal-setting and EQ have been shown to increase the ideal level of life. This result supports the findings of previous studies [23] that SFBT-based observational tasks increase the ideal level of life. The ideal level of life is also considered a relevant part of subjective fulfillment. This suggests that goal-setting and EQ are effective in increasing subjective fulfillment as perceived by the individual. However, TMQ and MQ did not have any additional effects on solution-building, positivity, or life idealism. Therefore, in terms of solution-building, positivity, and the ideal level of life, the effect of clarifying the solution image is limited, and the effect of goal-setting and EQ is considered to be significant.

The effects of each questioning technique were examined. However, it is important to note the possibility that the number of questions asked made a difference as it differed for each condition set in this study. For example, the perception of "I took the time to work on it" may have increased participants’ propensity to find benefits through adjustment for cognitive dissonance. On the other hand, if the number of questions was the only factor making a difference, one would expect to find differences between groups in a variety of variables as well. However, only certain variables showed significant differences in this study. Although the influence of the different numbers of questions cannot be ruled out, considering the results of this study and previous studies, the distinctive role of each questioning technique is considered to have been demonstrated. Nevertheless, it is important to control the number of questions and compare each questioning technique to examine the impact of the number of questions in detail.

## Conclusion

The results of this study showed that worksheets consisting of the TMQ, MQ, goal setting and EQ increased solution-building, positivity, time-oriented and an ideal level of life. In particular, since positivity and time-oriented are aspects of positive attitude towards life, it is possible to partially increase it by implementing the worksheet. As previously mentioned, worksheet-style support removes the constraints of time and place and allows people to work at their own pace [15]. Therefore, a worksheet consisting of the TMQ, MQ, goal setting and EQ is expected to be used as an easily accessible tool to improve the mental health of workers.

### Limitations of the present study and future research

This study has several limitations that should be considered when interpreting the findings. First, the participants in this study were working Japanese people between the ages of 20 and 39, who answered the questions online. It would be useful to replicate this study with young people, including middle and high school students, rather than with working adults. Second, although "time machine" is familiar to Japanese people owing to the influence of manga characters and is considered to be an effective framework for thinking about the long-term future, it may not be an effective framework in other cultures. If the term "time-machine" is not an effective framework for thinking about the long-term future, then the effectiveness of TMQ may be limited, and we need to carefully judge its effectiveness in other cultures. Third, no follow-up research has been conducted to examine the long-term effects of TMQ. Future research should include follow-up evaluation to determine the extent to which the observed short-term impacts are sustained. Such research would significantly develop the findings presented in this study. Fourth, each condition set in this study contained a different number of questions. To understand the differences in questioning techniques, it is important to determine whether the differences are due to the addition of questioning techniques or the increase in the number of questions. Therefore, it is important to examine the effects of the questioning technique with the number of questions controlled. Finally, although the sample size of this study was adequate in terms of power, differences in questioning techniques could be presented as smaller effect sizes, and such differences could not be tested. Therefore, it is important to ensure larger sample sizes and consider the differences in questioning techniques indicated by smaller effect sizes. Despite these limitations, this study extends empirical support and evidence for the use of TMQ in SFBT practice.

## Supporting information

S1 Data (XLSX)
